# Recent variant discoveries and emerging genetic mechanisms in autoinflammatory diseases

**DOI:** 10.3389/fimmu.2026.1910164

**Published:** 2026-09-15

**Authors:** Jinyun Chen, Shiliang Zhou, Ting Xu, Min Wu

**Affiliations:** Department of Immunology and Rheumatology, the 3rd Affiliated Hospital of Soochow University, Jiangsu, China

**Keywords:** autoinflammatory diseases (AID), functional validation, genetic testing strategy, molecular pathway, somatic mosaicism, variant

## Abstract

Autoinflammatory diseases (AIDs) comprise a heterogeneous group of disorders caused by dysregulated innate immune responses. Over the past decade, advances in next-generation sequencing have markedly expanded the spectrum of disease-associated genes and revealed novel genetic mechanisms, including somatic mosaicism and non-coding variation, while also highlighting the potential contribution of multilocus rare-variant burden and modifier effects. However, the rapid pace of gene discovery has also introduced new challenges in variant interpretation, disease classification, and clinical diagnosis. This review synthesizes findings from 100 publications published between 2014 and 2026 and summarizes recent genetic advances according to the major molecular pathways involved in autoinflammatory diseases. We review newly identified disease genes and pathogenic variants affecting ubiquitination and NF-κB regulation, inflammasome activation, type I interferon signaling, CDC42-mediated immune regulation, proteasome dysfunction, programmed cell death, cytokine signaling, and other emerging pathways. We further discuss recent insights into genotype–phenotype correlations, low-level somatic mosaicism, atypical gain- and loss-of-function variants, and multilocus rare-variant burden, including possible oligogenic or modifier effects. In addition, we summarize advances in genetic testing strategies, the increasing role of functional validation in interpreting variants of uncertain significance, and current challenges in establishing disease causality for newly proposed candidate genes. Finally, we compare the strength of evidence supporting recent genetic discoveries, highlight areas of ongoing controversy, and discuss future directions for improving molecular diagnosis and precision medicine in autoinflammatory diseases. By integrating recent discoveries within a pathway-based framework, this review provides an updated and clinically relevant overview of the rapidly evolving genetic landscape of AIDs.

## 1 Introduction

The concept of autoinflammation emerged just over two decades ago with the discovery that periodic fever syndromes are driven by innate immune dysregulation rather than adaptive immune dysfunction or autoantibodies. Since then, the field has expanded from a handful of monogenic recurrent fevers – familial Mediterranean fever, TNF receptor-associated periodic syndrome, cryopyrin-associated periodic syndromes, and mevalonate kinase deficiency – to encompass more than 50 distinct disorders affecting diverse pathways including ubiquitination, inflammasome assembly, type I interferon signaling, ER-Golgi trafficking, and necroptosis (1–5).

This rapid expansion has been driven primarily by advances in next-generation sequencing. Targeted gene panels, whole-exome sequencing, and whole-genome sequencing have enabled the discovery of novel genes, atypical phenotypes, and unexpected mechanisms. Among the most paradigm-shifting revelations has been the recognition that somatic mosaicism – postzygotic variants present in only a subset of cells – underlies many adult-onset autoinflammatory diseases previously considered idiopathic, including late-onset CAPS (6, 7) and VEXAS syndrome (2). Non-coding variants in genes such as *UBA1* have further expanded the diagnostic landscape beyond exonic regions (8, 9).

Despite these advances, significant challenges remain. Many patients with strong clinical features of autoinflammation lack a molecular diagnosis. Variants of uncertain significance (VUS) accumulate as sequencing becomes more widespread, necessitating functional validation. Oligogenic inheritance, where multiple rare variants may collectively modify disease expression, complicates traditional Mendelian models (10). Disagreements persist regarding the clinical significance of low-level mosaicism, the optimal first-line genetic test, and the standardization of functional assays.

This review synthesizes findings from 100 recent studies (2014–2026), organized according to major molecular pathways involved in autoinflammatory diseases. Within each pathway, we summarize recent genetic discoveries, highlight emerging disease mechanisms, compare the strength of current evidence, and discuss their diagnostic and clinical implications. **Tables 1**–**3** provide concise overviews of newly identified genes, disease phenotypes, and diagnostic advances, whereas Section 11 compares the current strength of evidence across recently described disorders. The final sections discuss unresolved challenges, limitations of current knowledge, and future directions for genetic diagnosis and precision medicine in autoinflammatory diseases.

**Table 1:** Genetic and mechanistic findings in autoinflammatory diseases categorized by evidence level.

Pathway	Gene(s)	Variant/inheritance pattern	Reported patients/families	Main mechanism	Evidence type	Replication	Functional validation	Variant interpretation/evidence status	Main phenotype(s)	Clinical actionability	Reference
Ubiquitination (E1)	***UBA1***	Somatic p.Met41 and non-coding/intronic variants; acquired mosaicism	Multiple cohorts; 301 VEXAS-suspected patients screened in one study, with 7 intronic variants identified	Loss of cytoplasmic UBA1 isoform and impaired ubiquitylation	Multiple clinical cohorts + mechanistic studies	**Replicated** for canonical p.Met41 variants; non-coding variants supported by limited reports	Yes; molecular and transcript-level evidence for selected variants	**Established** for canonical VEXAS variants; **emerging** for non-coding variants	VEXAS: vacuoles, cytopenia, chondritis, vasculitis	**Established**; diagnostic testing clinically relevant	(2, 8, 9, 11)
Ubiquitination (DUB)	***OTULIN***	Biallelic LOF; dominant-negative; haploinsufficiency	Multiple unrelated patients/families; one study reported 6 unrelated heterozygous patients	Biallelic LOF causing ORAS; dominant-negative and haploinsufficiency mechanisms expanding the phenotype	Genetic + functional + mechanistic evidence	**Replicated across multiple reports**, with mechanism-specific evidence	Yes; linear ubiquitin accumulation, LUBAC degradation, cell-death and inflammatory assays	**Established for major mechanism-specific phenotypes**; individual variants require variant-level assessment	Panniculitis, abscesses, pyoderma gangrenosum, infections, staphylococcal necrosis	Clinically relevant; mechanism-specific treatment implications	(17–23)
Ubiquitination (LUBAC)	***SHARPIN***	Biallelic LOF	2 individuals reported	Reduced NF-κB signaling and increased cell-death susceptibility	Single report + functional/mechanistic evidence	**Limited; single reported family/individual set**	Yes; functional evidence reported in affected individuals	**Emerging/limited genetic evidence**	Autoinflammation without dermatitis	Potentially actionable; evidence limited	(24)
NF-κB regulation	***TNFAIP3***	Heterozygous LOF; selected missense variants	Multiple families and diagnostic cohorts	A20 haploinsufficiency; impaired deubiquitination or increased proteasomal degradation	Multiple cohorts + functional studies	**Replicated**	Yes; NF-κB reporter and mechanistic assays	**Established gene–disease relationship**; individual missense variants require integrated interpretation	Behçet-like disease, early-onset systemic inflammation	**Clinically actionable**	(1, 12–16)
NF-κB transcription	***NFKB1***	Truncating monoallelic LOF	6 families in the principal study	Impaired autophagy with combined inflammasome/IFN activation	Familial genetic evidence + patient-cell functional evidence	**Replicated in multiple families in the reported cohort**	Yes; macrophage studies	**Established/well supported**	Necrotizing fasciitis, soft-tissue inflammation	Clinically relevant	(25)
NF-κB scaffold	***IKBKG***	X-linked variants; exon-5 skipping/splice variants	One principal X-linked syndrome plus additional splice-variant reports	NEMO-Δex5, IKKi stabilization, NF-κB and type I IFN activation	Genetic + mechanistic evidence	**Limited to emerging replication**	Yes; molecular and pathway activation studies	**Established for NDAS-associated variants; emerging for additional splice variants**	NDAS, autoinflammation with IFN signature	Clinically relevant	(26, 27)
Inflammasome	***NLRP3***	Germline GOF or somatic mosaic variants	Multiple cohorts; systematic functional study included 34 variants	Inflammasome GOF with variant-specific priming/activation requirements	Multiple genetic cohorts + extensive functional studies	**Multiple independent reports/cohorts**	Yes; caspase-1, ASC-speck and cytokine assays	**Established gene–disease relationship**; variant-level interpretation should integrate functional and genetic evidence	CAPS spectrum: urticaria, fever, hearing loss	**Established diagnostic and therapeutic relevance**	(29–41)
Inflammasome	***NLRC4***	Germline or somatic GOF variants	Multiple families/cases; low-level mosaic cases reported	Constitutive inflammasome activation; altered LRR oligomerization	Multiple case reports/families + functional studies	**Replicated**, although low-level mosaicism remains less extensively characterized	Yes; inflammasome/caspase-1 and experimental models	**Established for major NLRC4-AID phenotypes; low-level mosaic variants require careful interpretation**	FCAS4, MAS, late-onset fever, SLE-like presentation	Clinically relevant; IL-18 and inflammasome-directed management may inform care	(42–48)
Inflammasome	***NLRP1***	Biallelic, *de novo*, and segregating GOF variants	Multiple families/cases, including two cousins, a *de novo* case, a three-generation family, and two sisters	Inflammasome activation and ASC-speck formation	Genetic + patient-cell functional evidence	**Replicated across unrelated families/cases**	Yes; IL-1β, IL-18 and ASC-speck assays	**Established/well supported**, but individual variants require integrated classification	NAIAD: arthritis, dyskeratosis, corneal and mucosal lesions	Clinically relevant	(49–51)
Inflammasome adapter	***PSTPIP1***	Multiple rare variants; inheritance varies by phenotype	161 patients; 39 reported variants evaluated in one study; additional families/cases	Increased PSTPIP1–pyrin interaction and downstream inflammasome activation	Cohort-level variant analysis + selected functional studies	**Replicated phenotype; substantial variant-level uncertainty**	Yes for selected variants	**Variant-level interpretation heterogeneous; only a subset of reported variants supported as pathogenic**	PAPA, PAMI, FMF/MKD-overlap phenotypes	Clinically relevant, but variant-specific actionability depends on evidence	(52–54)
Caspase	***CASP1***	Heterozygous variant	Single reported patient in the cited study	Increased inflammasome activation and IL-1β/IL-18 signaling	Single case + functional validation	**Single reported case**	Yes; patient PBMC stimulation and inflammasome assays	**Proposed/limited evidence; functional support does not independently establish pathogenicity**	sJIA, recurrent MAS	Investigational	(55)
Interferon (cGAS–STING)	***TMEM173***	GOF variants	Multiple affected patients/families reported in the established disease spectrum	Constitutive STING activation	Genetic + patient-cell functional evidence	**Replicated**	Yes; constitutive IFNB1 transcription and STAT1 phosphorylation	**Established gene–disease relationship**	SAVI: vasculopathy, lung disease, systemic inflammation	**Clinically relevant**; JAK inhibition may be considered	(3)
Interferon (TBK1)	***TBK1***	Homozygous LOF	4 patients in the principal report	TNF-driven cell death/necroptosis	Genetic + patient-cell/mechanistic evidence	**Limited; principal report involved 4 patients**	Yes	**Well supported but relatively rare**	Chronic autoinflammation without severe viral susceptibility	Clinically relevant	(56)
Interferon (ARF1)	***ARF1***	Pathogenic variants reported in interferonopathy spectrum	Limited reports; exact number not specified in current manuscript	Defective GTPase function, mtDNA release and impaired STING recycling	Genetic + mechanistic studies	**Emerging; independent replication remains limited**	Yes; cellular STING/trafficking studies	**Emerging/proposed**	Type I interferonopathy	Investigational	(57, 58)
Interferon (REXO2)	***REXO2***	Dominant-negative variants	Limited reports; exact number not specified in current manuscript	mtRNA accumulation leading to MDA5 activation	Genetic + functional/mechanistic evidence	**Limited/emerging**	Yes	**Emerging/proposed**	Rash, eosinophilia, elevated IgE	Investigational	(59)
Interferon (COPA)	***COPA***	Dominant variants affecting C-terminal region	Multiple reports; exact number not specified in current manuscript	COPI dysfunction and ER stress	Genetic + functional/mechanistic evidence	**Replicated disease association**	Yes	**Established gene–disease relationship; individual variants require classification**	Autoinflammation and autoimmunity	Clinically relevant	(4)
Proteasome	***PSMB8/10, PSMA5, PSMC5***	Predominantly biallelic proteasome-related variants	Multiple reports across PRAAS/CANDLE spectrum; exact numbers not specified in current manuscript	Impaired proteasome assembly/expression and type I IFN activation	Genetic + pathway/functional evidence	**Replicated across proteasome-associated syndromes**	Yes for selected genes/variants	**Established for major PRAAS mechanisms; variant-level evidence varies**	PRAAS/CANDLE, interferon-driven inflammation	Clinically relevant	(60)
Small GTPase	***CDC42***	C-terminal variants	Multiple cases reported; exact number not specified in current manuscript	Golgi trapping with altered pyrin/STING signaling	Genetic + functional cellular studies	**Limited/emerging**	Yes; subcellular localization studies	**Emerging; selected C-terminal variants supported by functional data**	NOCARH: cytopenia, HLH, rash	Potentially clinically relevant	(62–67)
Mevalonate pathway	***PMVK***	Biallelic LOF	Limited patients/cases; exact number not specified in current manuscript	IL-1β hypersecretion	Genetic + functional evidence	**Limited/emerging**	Yes	**Emerging/proposed**	HIDS-like disease, arthritis, cytopenia	Investigational	(68–70)
Necroptosis	***RIPK1***	Cleavage-resistant variants	Multiple affected individuals reported; exact number not specified in current manuscript	Resistance to caspase-8 cleavage and constitutive necroptosis	Genetic + structural + *in vitro* functional evidence	**Replicated CRIA phenotype; variant-level evidence varies**	Yes; cleavage and necroptosis assays	**Established CRIA mechanism for reported variants**	CRIA syndrome	Clinically relevant	(71)
Necroptosis	***RIPK3***	Heterozygous GOF variant	One three-generation family in the cited study	Enhanced kinase activity, RIPK3 self-association, MLKL phosphorylation and necroptosis	Single family + patient-cell functional validation	**Single-family report**	Yes; patient PBMCs and pharmacological inhibition	**Proposed/emerging; requires independent replication**	Still’s disease-like autoinflammation	Investigational	(5)
IL-1 receptor	***IL1R1***	*De novo* variant	Single patient in the cited report	Selective impairment of IL-1Ra binding with preserved IL-1α/IL-1β binding, resulting in unopposed IL-1 signaling	Single case + biochemical and animal-model evidence	**Single reported case**	Yes; ligand-binding studies and mouse model	**Proposed/emerging; functional evidence supports mechanism but does not alone establish pathogenicity**	CRMO, unopposed IL-1 signaling	Potential therapeutic relevance; clinical evidence limited	(72)
Kinase signaling	***JAK1, LYN, IKBKB, IRAK2***	Predominantly GOF variants; gene-specific inheritance	Limited individual reports/families; exact numbers not specified in current manuscript	NF-κB and/or IFN pathway hyperactivation	Genetic + functional studies	**Limited/emerging and gene-specific**	Yes for selected variants	**Emerging; variant-level evidence heterogeneous**	Keratinization disease, vasculitis, sJIA and immune dysregulation	Investigational/gene-specific	(73, 74, 77–79)
Phospholipase	***PLCG2, PLCG1***	GOF or LOF variants; gene-specific inheritance	Multiple reports across PLAID/APLAID and related phenotypes	B-cell/T-cell signaling dysregulation	Genetic + functional studies	**Replicated across established PLCG2-associated phenotypes; PLCG1 evidence more limited**	Yes for selected variants	**Established for major PLCG2-associated disorders; emerging for newer PLCG1 mechanisms**	PLAID/APLAID, immune dysregulation, immunodeficiency	Clinically relevant but variant-specific	(75, 76)

Bold text highlights key evidence statements in the table, particularly regarding replication, variant interpretation/evidence status, and clinical actionability.

**Table 2:** Somatic mosaicism in autoinflammatory diseases: variant burden, cellular distribution, and detection characteristics.

Gene	Variant type	VAF range in blood	Cell populations/tissue distribution evaluated	Negative or unaffected populations reported	Associated phenotype	Detection method	Reported sequencing depth/analytical sensitivity	References
*NLRP3*	Somatic mosaicism	2–35%	Predominantly myeloid-restricted in late-onset disease; broader distribution reported in early-onset disease	Not consistently specified across studies	CAPS spectrum: urticaria, fever, hearing loss	Amplicon-based high-depth NGS	**>1500×** in studies reporting detection of variants at approximately 2–3% VAF; analytical sensitivity otherwise study-dependent	(6, 7, 29–34)
*UBA1*	Somatic and non-coding variants	10–90%	Myeloid-restricted distribution reported; specific myeloid populations varied by study	Not consistently reported in the current evidence base	VEXAS: vacuoles, cytopenia, chondritis, vasculitis	Exon-3 Sanger sequencing or NGS; broader sequencing for non-coding variants	Study- and platform-dependent; minimum validated VAF not consistently reported	(8, 9)
*NLRC4*	Somatic mosaicism	2–5%	Myeloid-restricted distribution reported	Specific negative populations not consistently reported	Late-onset fever, arthritis, rash; MAS not consistently present	ddPCR and high-depth NGS	Study-dependent; depth and validated lower limit of detection not consistently reported	(46–48)
*TNFRSF1A*	Somatic mosaicism	14%	B cells, neutrophils, and NK cells	Broad distribution rather than myeloid restriction was reported	Adult-onset TRAPS	High-depth NGS	Study-dependent; validated analytical sensitivity not consistently reported	(28)

Bold text highlights key evidence statements in the table, particularly regarding replication, variant interpretation/evidence status, and clinical actionability.

**Table 3:** Functional validation assays and their role in variant interpretation.

Gene	Experimental system	Assay type	Primary readout	Functional interpretation	Role in variant interpretation	References
*TNFAIP3*	Cellular NF-κB reporter system; patient-derived cells where available	NF-κB luciferase reporter assay	Fold change in NF-κB activation following TNF/LPS stimulation	Increased NF-κB activity supports impaired A20-mediated negative regulation	Provides functional evidence that may support pathogenicity of selected variants when integrated with genetic, clinical, and population evidence	(13)
*NLRP3*	Cellular inflammasome models; patient-derived cells in selected studies	Caspase-Glo 1 assay	Caspase-1 activity	Increased caspase-1 activity supports NLRP3 inflammasome GOF	Supports functional assessment of NLRP3 variants; does not independently establish pathogenicity	(97)
*NLRP3*	Cellular inflammasome models	ASC-speck formation by flow cytometry	Percentage of ASC-positive cells	Increased ASC-speck formation supports enhanced inflammasome assembly	Provides mechanistic evidence for altered NLRP3 function and can contribute to variant interpretation	(41)
*NLRP3*	Cellular inflammasome models/stimulated cells	IL-1β and IL-18 secretion by ELISA	Cytokine concentration in culture supernatant	Increased IL-1β/IL-18 production supports inflammasome hyperactivation	Provides complementary functional evidence for *NLRP3* GOF; interpretation should be integrated with other ACMG/AMP evidence	(41)
*PSMB9* *(PRAAS)*	Patient-derived cells/cellular interferon-response system	Interferon-signature analysis by ddPCR	Expression of MX1, ISG15, IFIT1, GBP1, and IRF1	Increased interferon-stimulated gene expression supports activation of the type I IFN pathway	Supports the biological mechanism of selected variants; does not by itself establish pathogenicity or determine treatment response	(61)
*NLRP12*	Patient PBMCs	Cytokine profiling following LPS/MDP stimulation	IL-1β, TNF-α, IL-17, and IL-10 concentrations	Altered cytokine responses support abnormal inflammatory signaling	May help characterize functional consequences of variants but should be interpreted as supportive rather than definitive evidence	(98)
*OTULIN*	Patient-derived cells/cellular ubiquitination system	Linear ubiquitin accumulation by Western blot	M1-linked polyubiquitin levels	Increased linear ubiquitin accumulation supports impaired OTULIN deubiquitinase activity	Provides functional evidence for loss of DUB function and may contribute to variant classification when assay validity and other evidence are adequate	(19, 20)
*CDC42*	Patient-derived cells or cellular localization system	Subcellular localization by immunofluorescence	Golgi versus cytoplasmic distribution	Abnormal Golgi trapping supports altered CDC42 trafficking associated with pathogenic C-terminal variants	Supports a mechanistic effect of selected variants; should be integrated with phenotype, segregation, and other genetic evidence	(65)

For key mechanistic claims, we preferentially indicate the experimental system, principal functional assay or readout, and direction of the observed effect when these details are available in the primary studies. Because the amount and maturity of functional evidence vary substantially among pathways, the depth of individual sections reflects both the strength and impact of the available evidence rather than providing equal methodological detail for every disease entity.

Throughout this review, the strength of genetic and mechanistic evidence is reflected explicitly in the terminology used to describe disease associations. Established gene–disease relationships supported by multiple independent cohorts, replicated case series, consistent segregation, and/or well-validated functional evidence are described using terms such as “established,” “pathogenic,” or “causes.” Associations supported primarily by a single family, a single case, or limited functional evidence are described more cautiously as “associated with,” “reported in,” “proposed to contribute to,” or “supported by functional data.” The terms “new disease,” “first disease,” and equivalent priority claims are reserved for findings for which the available genetic, clinical, and functional evidence is sufficiently established. Functional evidence alone is not considered sufficient to establish pathogenicity and is interpreted together with genetic, population, clinical, segregation, and disease-validity evidence.

### 1.1 Literature search strategy

This narrative review evaluated recent genetic, clinical, mechanistic, and diagnostic advances in systemic autoinflammatory diseases. Literature searches were conducted in PubMed/MEDLINE, Web of Science Core Collection, and Scopus for publications published from January 1, 2014, through March 31, 2026. The complete database-specific search strategies, search dates, and applied filters are provided in **Supplementary Table 1**.

The search strategy combined terms related to autoinflammatory disease with genetic variation, somatic mosaicism, gain- and loss-of-function mechanisms, oligogenic or multilocus variation, functional validation, and genetic testing. In addition to the core searches, targeted searches were performed using the names of individual genes, pathways, and syndromes discussed in this review. Reference lists of relevant primary studies and selected reviews were screened manually to identify additional publications that met the review scope.

Eligible publications included original research articles, cohort studies, case series, case reports, genetic studies, and mechanistic or functional studies that provided relevant human genetic, clinical, cellular, biochemical, or animal-model evidence. Publications were excluded when they were unrelated to systemic autoinflammatory disease or did not provide information relevant to genetic mechanisms, variant interpretation, functional biology, somatic mosaicism, or genetic diagnosis. Publications not available in English were excluded.

Conference abstracts were considered eligible when they contained directly relevant primary data and no corresponding full-text peer-reviewed publication was identified within the review period. Preprints were retained only when they provided directly relevant primary data and no peer-reviewed version was identified by the end of the search period. Review articles, consensus statements, and clinical guidelines were not treated as primary evidence but were used for background context and reference-list screening.

Because this was a narrative review rather than a systematic review, study selection was based on relevance to the predefined scope, novelty, mechanistic informativeness, strength and replication of the available evidence, and clinical or diagnostic relevance. Greater emphasis was placed on replicated findings and mechanistically informative primary studies, while single-family, single-case, conference-abstract, or preprint reports were retained only when they provided relevant emerging evidence. A total of 100 publications were included in the final narrative synthesis.

### 1.2 Evidence and methodological reporting

To facilitate interpretation of the primary evidence, we report key methodological features when they are available from the original studies, particularly for low-level mosaicism and functional variant validation. For mosaic variants, we indicate the sequencing or detection approach, approximate sequencing depth or analytical sensitivity when reported, variant allele frequency (VAF), and the cellular populations in which mosaicism was detected or not detected. For functional studies, we specify the experimental system and the principal assay or readout supporting the proposed mechanism. When variants are reclassified, we indicate the classification framework and the principal evidence supporting the classification when these details are available. Where the primary publication does not provide sufficient methodological information, we avoid inferring experimental parameters and explicitly distinguish reported findings from methodological details that remain unavailable.

### 1.3 Evidence synthesis strategy

Rather than presenting each primary study as an isolated observation, we synthesize findings across independent cohorts according to shared molecular mechanisms, inheritance patterns, variant classes, and clinical implications. Recurrent findings supported by multiple independent studies or cohorts are distinguished from observations based primarily on single families or individual cases. Where studies provide apparently divergent results, we compare the experimental systems, variant classes, cellular contexts, and clinical phenotypes before proposing a unifying interpretation. This approach is intended to emphasize reproducible biological principles while preserving uncertainty where the available evidence remains limited.

## 2 Ubiquitination and NF-κB regulation

The ubiquitin system plays a central role in regulating innate immune signaling by controlling protein stability and NF-κB activation. Ubiquitination-related autoinflammatory diseases illustrate how disruption at different levels of the ubiquitin system can converge on excessive inflammatory signaling. The available evidence can be broadly grouped into three mechanisms: somatic disruption of ubiquitin activation, loss of negative regulation of NF-κB signaling, and altered ubiquitin editing within the LUBAC pathway. These mechanisms differ in inheritance and cellular context but share a common consequence of impaired control of inflammatory responses.

### 2.1 *UBA1*: somatic mosaicism and VEXAS syndrome

*UBA1*-associated VEXAS is a paradigmatic example of somatic mosaicism causing adult-onset autoinflammation. The recurrent p.Met41Leu/Thr/Val variants impair production of the cytoplasmic UBA1b isoform and promote expression of a catalytically impaired alternative isoform, resulting in defective ubiquitylation and inflammatory activation (2, 11). Among the canonical p.Met41 substitutions, p.Met41Val has been associated with poorer survival and more severe disease, consistent with a stronger effect on UBA1b translation (11).

The spectrum of pathogenic *UBA1* variation extends beyond exon 3. In a cohort of 301 VEXAS-suspected patients who were negative for exon-3 mutations, Sirenko et al. (8) identified seven patients with intronic variants, including a recurrent variant with a median VAF of 78% (range, 38–97%) in peripheral blood. These variants were reported as myeloid-restricted and were associated with reduced *UBA1* transcript and protein levels in CD14+ monocytes. Cardona et al. (9) independently identified a splice-site variant causing aberrant splicing and multiple in-frame transcripts. Together, these observations indicate that the diagnostic spectrum of VEXAS includes both canonical somatic missense variants and non-coding or splice-altering variants, supporting analysis beyond exon 3 when clinical suspicion remains high despite negative testing (8, 9).

The appropriate testing strategy depends on the suspected variant mechanism and the laboratory’s validated analytical performance. Deep targeted sequencing may be advantageous for detecting low-level somatic variants in a predefined region, whereas WGS may provide broader assessment of intronic and regulatory variation. For suspected splice-altering variants, RNA analysis from an appropriate tissue or cell population can provide important evidence of aberrant splicing when technically feasible. Because the sensitivity of mosaic variant detection depends on sequencing depth, error-correction methods, specimen type, and variant allele fraction, a coverage threshold such as ≥1500× should be regarded as platform- and laboratory-dependent rather than as a universal clinical requirement.

Accordingly, the presence of an intronic *UBA1* variant should not be considered sufficient by itself to establish VEXAS. Interpretation should integrate the clinical phenotype, variant allele fraction, population frequency, predicted or demonstrated splicing effect, and, where available, RNA evidence and independent replication.

### 2.2 *TNFAIP3*: A20 haploinsufficiency and NF-κB dysregulation

*TNFAIP3*-related autoinflammation is predominantly characterized by impaired negative regulation of NF-κB. Heterozygous loss-of-function variants cause HA20, typically presenting with early-onset Behçet-like or systemic inflammation (1). The expanding spectrum of missense variants demonstrates that reduced A20 abundance, impaired deubiquitination, and altered interactions with inflammatory signaling proteins can produce similar clinical phenotypes through different molecular mechanisms (12–15).

Functional studies also illustrate why genotype alone is insufficient for interpreting *TNFAIP3* missense variants. Khouri et al. (12) showed that p.(Leu236Pro) reduces A20 abundance through enhanced proteasomal degradation and impairs TRAF6 deubiquitination, whereas Kadowaki et al. (13) found that only one of three evaluated missense VUS, p.Glu192Lys, impaired NF-κB reporter activity. Other variants have been linked to NF-κB overactivation or increased NLRP3 responsiveness (14, 15). Thus, the most consistent evidence supports loss of A20-mediated inflammatory control, whereas the pathogenicity of individual missense variants requires functional evaluation rather than inference from variant location alone. In a diagnostic cohort, monogenic HA20 accounted for 16% of suspected Behçet disease cases, further supporting consideration of *TNFAIP3* testing in phenotypically atypical patients (16).

### 2.3 *OTULIN*: distinct inheritance mechanisms converging on ubiquitin editing

*OTULIN*-associated disease provides an instructive example of how different inheritance patterns can disrupt the same ubiquitin-editing pathway. Biallelic loss-of-function variants cause *OTULIN*-related autoinflammatory syndrome (ORAS), characterized by panniculitis, fever, diarrhea, and arthritis, whereas dominant-negative variants and haploinsufficiency expand the phenotype toward ORAS-like disease or infection- and trauma-associated tissue necrosis (17–23).

The functional consequences differ according to the variant mechanism. Biallelic deficiency disrupts OTULIN-dependent removal of M1-linked ubiquitin chains and alters LUBAC/TNF signaling, while dominant-negative variants are associated with linear ubiquitin accumulation (17, 19, 20). Heterozygous variants identified in multiple unrelated patients produced a distinct susceptibility to severe staphylococcal or trauma-associated necrosis, supporting haploinsufficiency as a separate disease mechanism (21). Additional biallelic variants have broadened the phenotype to sterile abscesses and pyoderma gangrenosum, with the latter associated with NLRP3 hyperactivation and increased keratinocyte TNF sensitivity (18, 22, 23). Thus, OTULIN disease is better viewed as a spectrum determined by allelic mechanism rather than as a single phenotype associated with one mode of inheritance.

### 2.4 *SHARPIN, NFKB1, and IKBKG*: altered NF-κB control with distinct inflammatory outputs

*SHARPIN, NFKB1*, and *IKBKG* further demonstrate that disruption of NF-κB regulation can produce clinically distinct autoinflammatory phenotypes depending on the affected molecular component. Biallelic *SHARPIN* loss of function causes autoinflammation without the severe dermatitis predicted from animal models, whereas monoallelic NFKB1 loss of function and IKBKG abnormalities produce distinct inflammatory syndromes (24–27).

The available functional evidence suggests that these disorders do not represent equivalent forms of NF-κB deficiency. SHARPIN deficiency is associated with attenuated canonical NF-κB signaling and increased susceptibility to inflammatory cell death; NFKB1 deficiency has been linked to impaired autophagy together with inflammasome and type I interferon activation; and the *IKBKG* exon-5-skipping phenotype involves NEMO-Δex5 and combined NF-κB/type I interferon activation (24–27).Thus, the shared pathway-level label of “NF-κB dysregulation” encompasses both impaired and aberrantly activated signaling, with the clinical phenotype determined by the specific molecular lesion and its downstream cellular consequences.

## 3 Inflammasomopathies

Inflammasomes are multiprotein complexes that detect cellular stress and microbial danger signals, leading to caspase-1 activation, maturation of IL-1β and IL-18, and pyroptotic cell death. Over the past decade, advances in genomic sequencing have substantially expanded the spectrum of inflammasome-related autoinflammatory diseases, revealing novel pathogenic mechanisms such as low-level somatic mosaicism, atypical gain-of-function variants, and variable genotype–phenotype correlations. Somatic mosaicism is particularly important in *NLRP3-* and *NLRC4-*associated disease and may contribute to late-onset or atypical presentations. It has also been described in genes outside the inflammasome pathway, such as *TNFRSF1A* in adult-onset tumor necrosis factor receptor-associated periodic syndrome (TRAPS), emphasizing that the affected cellular compartments and detectable VAFs may differ across genes (28). **Table 2** summarizes representative mosaicism studies across autoinflammatory diseases, allowing comparison of these methodological and clinical features.

VAF values represent the ranges reported in the cited studies and should not be interpreted as universal diagnostic thresholds. “Myeloid-restricted” is used only when mosaicism was reported predominantly or exclusively in myeloid-lineage cells; the specific cell populations examined were not uniformly reported across studies. Where individual myeloid populations or negative cell populations were not specified in the primary reports available for this review, this is indicated as “not consistently reported” rather than inferred. Sequencing depth and analytical sensitivity are study-, platform-, specimen-, and laboratory-dependent. A reported depth of >1500× for detection of approximately 2–3% VAF therefore represents the performance of the cited assay rather than a universal clinical requirement. Detection of low-level mosaicism should additionally consider error rates, technical replicates, orthogonal confirmation, specimen type, and the validated limit of detection of the individual laboratory assay.

### 3.1 *NLRP3*: from germline mutations to low-level somatic mosaicism and atypical LRR variants

*NLRP3*-associated autoinflammation is best understood as a spectrum of gain-of-function mechanisms rather than a single genotype–phenotype pattern. Germline variants in the NACHT and LRR regions can produce constitutive or partially stimulus-dependent inflammasome activation, whereas somatic mosaicism introduces an additional layer of variation determined by variant allele frequency, developmental timing, and hematopoietic lineage distribution.

Somatic mosaicism is now a reproducible feature of *NLRP3-*associated disease, particularly in patients without an identifiable germline *NLRP3* variant with late-onset or atypical CAPS. Independent cohorts have reported mosaic variants in approximately 12.5–40% of previously mutation-negative CAPS populations, with VAFs ranging from approximately 2% to 35% in blood. High-depth (1500×) targeted sequencing has been important for identifying low-level variants, including variants at 2–3% VAF. However, the biological distribution of mosaicism is heterogeneous. Early-onset cases tend to show broader and more stable distribution across tissues, whereas late-onset disease is more often associated with myeloid enrichment and, in some patients, progressive increases in VAF over time. These observations suggest that developmental timing and clonal expansion, rather than VAF alone, contribute to the clinical phenotype (6, 7, 29–34).

The clinical phenotype of *NLRP3* variants also depends on the affected domain and the degree of inflammasome activation. The systematic characterization of *NLRP3* variants has identified functionally distinct groups according to their requirements for priming and activation signals. In particular, atypical LRR-domain variants may retain a requirement for an activation signal despite producing clinically relevant inflammation, resulting in phenotypes that differ from classic CAPS and can be associated with diagnostic uncertainty (35–41). Across independent reports, LRR variants have been associated with less typical cold-induced urticaria but increased hearing involvement or other organ-specific manifestations, indicating that variant location can modify rather than simply determine phenotype.

Taken together, the strongest replicated evidence supports three conclusions: first, *NLRP3* disease is predominantly driven by gain-of-function mechanisms; second, low-level somatic mosaicism is an important cause of late-onset and atypical disease and requires sufficiently sensitive sequencing; and third, variant domain and activation requirements contribute to phenotypic heterogeneity. In contrast, the clinical significance of extremely low-level mosaicism and the threshold at which a mosaic clone should be considered pathogenic remain unresolved, particularly when distinguishing pathogenic mosaicism from age-related clonal hematopoiesis.

### 3.2 *NLRC4*: from severe early-onset MAS to late-onset mosaicism and LRR oligomerization

*NLRC4*-associated autoinflammation is characterized predominantly by gain-of-function mechanisms that promote constitutive inflammasome activation, but the clinical spectrum varies substantially with variant location and genetic architecture. Germline variants affecting the NBD, NOD, or LRR regions have been associated with disease ranging from familial cold autoinflammatory syndrome to severe early-onset MAS. Across these reports, excessive caspase-1 activation and markedly increased IL-18 emerge as recurring functional and clinical features, with IL-18 particularly useful for distinguishing *NLRC4*-associated disease from some other inflammasomopathies (42–44).

The available functional studies suggest that different *NLRC4* domains can produce gain of function through distinct structural mechanisms. NBD/NOD variants can promote constitutive activation, whereas LRR-domain variants may alter oligomerization interfaces required for inflammasome assembly (42–44). A representative example is the LRR-domain p.Trp655Cys variant (c.1965G>C), reported in two unrelated patients with early-onset MAS, which affects the LRR–LRR oligomerization interface and was shown to enhance NLRC4 inflammasome activation (44). A subsequent report identified a different substitution at the same residue, p.Trp655Ser, in a patient with systemic lupus erythematosus and her son, who had neonatal lupus complicated by MAS; this single-family observation provides preliminary evidence of an association but has not been independently replicated (45). Together, these observations suggest that residue 655 may represent a functionally important hotspot within the *NLRC4* LRR domain associated with diverse inflammatory phenotypes. However, the limited number of reported patients and lack of independent genotype–phenotype replication preclude assigning a specific clinical phenotype to individual substitutions at this residue. Thus, the common pathogenic endpoint is excessive inflammasome activation, but the molecular route to activation and the resulting clinical phenotype may be variant-dependent.

Somatic *NLRC4* mosaicism extends this spectrum to later-onset disease. Independent reports have identified low-level mosaic variants with VAFs of approximately 2–6% in patients presenting with recurrent fever, gastrointestinal symptoms, arthritis, or systemic inflammation without the severe MAS phenotype characteristic of some germline variants (46–48). These observations suggest that the proportion and distribution of mutant cells may modify disease severity, although the small number of reported mosaic cases prevents firm genotype–VAF–phenotype correlations.

Overall, constitutive NLRC4 inflammasome activation is supported by multiple independent genetic and functional observations, whereas the specific relationships among variant domain, residue-level effects, mosaic VAF, cellular distribution, and clinical severity remain less well established. The p.Trp655Cys and p.Trp655Ser observations illustrate how different substitutions at the same LRR residue may be associated with distinct inflammatory phenotypes, but these observations remain insufficient to establish a deterministic residue–phenotype relationship. The distinction between replicated mechanisms and limited patient-level observations is therefore important when interpreting newly identified *NLRC4* variants.

### 3.3 *NLRP1*: linking inflammasome activation to skin dyskeratosis and arthritis

*NLRP1*-associated autoinflammatory disease is characterized by recurrent systemic inflammation accompanied by variable skin manifestations and arthritis. NAIAD (*NLRP1*-associated autoinflammation with arthritis and dyskeratosis) is characterized by early-onset autoinflammation accompanied by arthritis, dyskeratosis, and corneal involvement. Recent genetic studies have further expanded the molecular mechanisms underlying this syndrome.

While *NLRP3* and *NLRC4* were relatively well established, *NLRP1* remained less explored until Grandemange et al. (49) identified the first disease-associated *NLRP1* variants in NAIAD: a homozygous p.Arg726Trp in two cousins and a *de novo* p.Pro1214Arg in a Dutch girl, with elevated caspase-1 and IL-18. Herlin et al. (50) expanded the phenotype to include corneal intraepithelial dyskeratosis, mucosal inflammation, tooth abnormalities, and eczema, reporting a novel *NLRP1* p.Ala59Pro segregating with incomplete penetrance over three generations; patient PBMCs showed increased IL-1β and ASC speck formation. Li et al. (51) described a homozygous p.Leu813Pro in two sisters with different severities (keratoacanthomas vs. keratosis lichenoides chronica). The mutation activated the NLRP1 inflammasome, and the more severely affected sister carried additional variants associated with atopy and psoriasis, suggesting disease-modifying effects of secondary genetic variants.

### 3.4 *PSTPIP1*: variant pathogenicity and pyrin inflammasome dysregulation

Pathogenic *PSTPIP1* variants are associated with PAPA (pyogenic arthritis, pyoderma gangrenosum, acne) and PAMI syndromes, but the literature has been clouded by overinterpretation of rare variants. Boursier et al. (52) analyzed 161 patients and concluded that only 7/39 reported *PSTPIP1* variants are truly pathogenic (author-defined criteria), emphasizing the need for functional validation. Önen et al. (53) described a three-generation family with an FMF/MKD-overlapping phenotype without *MEFV*/*MVK* mutations, and identified a rare p.Arg228Cys variant that increased PSTPIP1-pyrin interaction and caspase-1/IL-1β in inflammation-induced patient samples. Takahashi et al. (54) reported a male infant with pancytopenia and a p.Asn236Lys variant, showing progressive increase in serum zinc and autoinflammation markers, supporting the PAMI diagnosis.

### 3.5 *CASP1*: a direct inflammasome component in sJIA and MAS

Jørgensen et al. (55) identified a heterozygous *CASP1* p.Arg161His variant in a patient with systemic JIA and recurrent MAS. Patient and control PBMCs were stimulated with 200 ng/ml LPS for 3 h followed by 10 µM nigericin for 1 h for IL-1β and IL-18 expression, and with 1 µg/ml LPS and 10 ng/ml TNFα for 20 h for IL-6 and CXCL10 expression. LPS stimulation of patient PBMCs induced high IL-6, and NLRP3 inflammasome activation increased IL-1β, IL-18, and caspase-1 activity. The variant also increased NF-κB activation in a RIP2-dependent manner, linking the inflammasome directly to upstream signaling.

Despite involving different inflammasome components, recent studies reveal several recurring mechanistic themes. First, low-level somatic mosaicism has emerged as an important cause of disease, particularly in patients previously considered mutation negative. Second, pathogenic variants increasingly involve regulatory domains rather than classical hotspot residues, expanding recognized genotype–phenotype correlations. Third, distinct variants within the same gene may produce remarkably different clinical manifestations, emphasizing the importance of functional characterization beyond simple variant identification.

## 4 Type I interferonopathies and trafficking defects

Type I interferonopathies increasingly encompass disorders that perturb different steps of nucleic-acid sensing, intracellular trafficking, and proteostasis. Across the newly described disorders, a recurrent theme is that constitutive or dysregulated interferon signaling can arise either from direct pathway activation or indirectly from defects in organelle homeostasis and nucleic-acid clearance. The resulting interferon signature therefore represents a shared downstream phenotype rather than evidence of a single molecular mechanism.

### 4.1 STING and *TBK1*: divergent mechanisms within interferon signaling

The clearest examples of direct pathway dysregulation are provided by gain-of-function variants in *TMEM173* (encoding STING) and loss-of-function variants in *TBK1*. STING GOF causes SAVI through constitutive activation of the cGAS-STING pathway, with patient cells showing increased IFNB1 transcription and STAT1 phosphorylation (3). In contrast, homozygous *TBK1* loss of function does not simply produce an equivalent interferon-deficient phenotype. Taft et al. (56) reported chronic autoinflammation without severe viral susceptibility, with impaired IFN-I induction but preserved IL-6 responses and evidence that TNF-induced necroptosis contributed to inflammation. Anti-TNF treatment ameliorated the clinical phenotype (56). This finding is mechanistically paradoxical because TBK1 is canonically a positive regulator of type I interferon production downstream of innate immune sensors. In this setting, however, TBK1 deficiency uncoupled impaired interferon induction from preserved inflammatory cytokine signaling, allowing TNF-driven necroptosis to become a dominant source of inflammation.

Together, these observations demonstrate that inflammatory disease can arise from both excessive activation and impaired signaling within the same broader interferon network. This distinction is clinically relevant because pathway inhibition must be directed at the dominant inflammatory output rather than inferred solely from the position of the genetic defect.

### 4.2 *ARF1*: trafficking and mitochondrial mechanisms converge on STING activation

*ARF1*-associated disease illustrates how intracellular trafficking defects can activate the interferon pathway without directly altering STING itself. Hirschenberger et al. (57) identified heterozygous GTPase-defective *ARF1* variants associated with both mitochondrial DNA release and impaired retrograde STING recycling. These mechanisms converge on persistent cGAS-STING activation and STING accumulation at the Golgi/ERGIC. A distinct *de novo* p.Arg19Cys variant described by Lang et al. (58) selectively impaired COPI binding and STING transport without affecting mitochondrial integrity, indicating that the trafficking component of ARF1 dysfunction can be sufficient to drive interferon activation.

The independent observations therefore support a common pathogenic endpoint—persistent STING signaling—but suggest that the relative contribution of mitochondrial integrity and STING trafficking is variant-dependent. The reduction of chronic IFN signaling by STING inhibitors in patient fibroblasts further provides proof-of-concept that the shared downstream pathway may represent a therapeutic target (58). These findings suggest that STING inhibition would be mechanistically most directly relevant to variants whose dominant defect is impaired STING recycling or constitutive downstream STING activation, whereas variants in which mitochondrial DNA release is a major upstream driver may not be fully corrected by STING blockade alone.

### 4.3 *REXO2 and COPA*: organelle homeostasis as an upstream determinant of interferon activation

*REXO2* and *COPA* provide complementary examples of how defective RNA surveillance and intracellular trafficking can generate interferon responses. The dominant-negative *REXO2* p.Thr132Ala variant impaired RNA cleavage and promoted mitochondrial dsRNA accumulation, providing a substrate for MDA5 sensing (59). In contrast, C-terminal *COPA* variants disrupted COPI-dependent trafficking and were associated with cGAS/STING-dependent type I interferon activation, ER stress, and NF-κB activation (4).

Although these genes act at different cellular processes, both findings support the broader principle that disruption of mitochondrial RNA handling or ER/Golgi trafficking can create endogenous nucleic-acid signals that sustain inflammatory responses. The COPA observations also extend the spectrum beyond the previously recognized N-terminal WD40-domain variants, suggesting that variant location can modify the mechanism of disease (4, 59).

### 4.4 Proteasome-associated autoinflammatory syndromes: proteostasis defects and interferon signatures

PRAAS/CANDLE represents another mechanistically distinct route to interferon activation. Papendorf et al. (60) identified variants affecting *PSMB8, PSMB10, PSMA5*, and *PSMC5*, with functional consequences involving proteasome expression or incorporation into the 26S proteasome. These findings expand the genetic spectrum beyond the canonical proteasome subunits while retaining a common biological theme of impaired proteostasis associated with type I interferon activation.

The *PSMB9* VUS reported by Merhar et al. (61) illustrates how this pathway-level signature can also have diagnostic and therapeutic relevance. Interferon-inducible gene profiling supported a pathogenic interpretation and helped guide ruxolitinib treatment. However, this single-patient observation should be distinguished from the broader, independently supported association between proteasome dysfunction and PRAAS.

### 4.5 Interferon signatures as a tool for resolving unclassifiable autoinflammation

In patients without a definitive molecular diagnosis, interferon signatures may help distinguish biologically different inflammatory states. De Jesus et al. (27) analyzed 66 undifferentiated SAID patients and identified several disease entities through interferon-related profiling, including IL-18-mediated pulmonary alveolar proteinosis with recurrent MAS, NEMO-NDAS, and *SAMD9L*-associated autoinflammatory disease. Importantly, some patients with *LRBA, IKBKG*, or *SAMD9L* variants showed increased inflammatory-regulated genes suggestive of prominent NF-κB rather than a pure type I interferon phenotype (27).

Thus, interferon profiling may be most useful not as a stand-alone diagnostic label but as a pathway-level discriminator that can guide interpretation of heterogeneous genetic findings. Across this section, the convergent evidence supports three major routes to interferonopathy: direct signaling dysregulation, trafficking/organelle defects, and impaired proteostasis or nucleic-acid surveillance.

## 5 Cytoskeletal and small GTPase pathways: CDC42

*CDC42*-associated autoinflammation illustrates how variants in a single small GTPase can produce different inflammatory phenotypes through convergent defects in intracellular trafficking. The most consistent clinical phenotype is NOCARH, characterized by neonatal or early-onset cytopenia, systemic inflammation, rash, and HLH, but the downstream inflammatory pathway appears to vary according to the affected region and biochemical consequence of the variant (62–67).

The recurrent p.Arg186Cys variant and other C-terminal *CDC42* variants provide the strongest evidence for a trafficking-centered mechanism. Independent functional studies have linked these variants to abnormal palmitoylation or intracellular localization, Golgi retention, and impaired vesicular trafficking (64–66). These defects can activate different inflammatory pathways: Golgi-trapped variants have been associated with pyrin inflammasome assembly, whereas impaired anterograde and retrograde trafficking can promote STING accumulation, type I interferon activation, and ER stress (65, 66). Mitochondrial abnormalities have also been reported, providing an additional potential source of inflammatory signaling (67).

Thus, the emerging model is not that all *CDC42* variants produce autoinflammation through a single downstream pathway, but that abnormal CDC42 localization and trafficking constitute a common upstream defect from which several inflammatory programs can arise. Clinical reports of responses to emapalumab, IL-1β inhibition, or ruxolitinib further suggest that the dominant downstream pathway may differ between patients, although treatment evidence remains limited to individual cases and small series (62, 63, 67).

## 6 Mevalonate pathway: PMVK deficiency

*PMVK* deficiency extends the genetic spectrum of mevalonate-pathway autoinflammation beyond the established *MVK*-associated disorders. Although only a small number of patients have been reported, the available cases show a recurring clinical pattern of recurrent fever, arthritis or other inflammatory manifestations, cytopenia, and biochemical features overlapping with HIDS/MKD (68–70).

The molecular and functional evidence is also directionally consistent across the reported variants. Berner et al. (68) described a homozygous p.Val131Ala variant associated with functionally confirmed *PMVK* deficiency, while Yıldız et al. (69) identified compound heterozygous variants in a child with an HIDS-like phenotype. Jairaman et al. (70) subsequently reported a homozygous p.Ala133Val variant and increased IL-1β production in patient mononuclear cells. Together, these observations support *PMVK* deficiency as a cause of mevalonate-pathway-associated inflammation and suggest that IL-1-mediated inflammation may contribute to the phenotype.

However, the evidence remains substantially less mature than for *MVK* deficiency. The reported cases are few, and independent genotype–phenotype correlations and standardized functional characterization are limited. *PMVK* should therefore currently be regarded as an emerging cause of HIDS-like autoinflammation rather than as a fully delineated phenotypic spectrum. The practical implication is that additional mevalonate-pathway genes may warrant evaluation in patients with convincing HIDS/MKD-like disease but no pathogenic *MVK* variant (68–70).

## 7 Necroptosis pathways: *RIPK1* and *RIPK3*

*RIPK1-* and *RIPK3*-associated autoinflammatory diseases provide genetic evidence that dysregulated programmed cell death can itself drive systemic inflammation. Although the pathogenic variants affect different components of the necroptotic pathway, both reported disorders converge on excessive or inadequately controlled necroptotic signaling rather than on a primary defect in pathogen sensing.

For *RIPK1*, CRIA is caused by variants affecting the caspase-8 cleavage site. Reula et al. (71) identified p.Leu321Arg and p.Asp324Gly variants and showed that structural changes reduced susceptibility to caspase-8-mediated cleavage, with functional studies supporting constitutive necroptotic activation. The mechanism therefore represents a failure of an endogenous checkpoint that normally restrains RIPK1-dependent cell death.

*RIPK3*-associated disease represents a complementary mechanism at a downstream node. Chen et al. (5) identified a heterozygous p.Gln134Lys variant associated with a Still’s disease-like phenotype. Functional studies showed enhanced kinase activity, RIPK3 self-association, MLKL phosphorylation, and necroptosis, together with increased NF-κB and MAPK signaling in patient PBMCs; pharmacological RIPK3 inhibition reduced the inflammatory signature (5).

Taken together, the two disorders support a common model in which excessive necroptosis can generate sterile systemic inflammation, while the specific molecular lesion determines whether disease arises from failure to restrain RIPK1 cleavage or direct enhancement of RIPK3 activity. The evidence is currently based on a limited number of genetic observations, so the broader genotype–phenotype spectrum and the frequency of necroptosis-driven autoinflammation remain to be established.

## 8 Other cytokine pathways and novel genes

Cytokine-mediated signaling coordinates communication between innate and adaptive immune cells. Both excessive activation and impaired regulation of cytokine receptors or downstream signaling molecules can result in autoinflammatory disease. This section summarizes recently identified disorders affecting cytokine signaling pathways and discusses their molecular and clinical characteristics.

The emerging genes discussed in this section converge on several signaling nodes rather than representing unrelated disorders. *IL1R1, IRAK2*, and *IKBKB* primarily alter IL-1/NF-κB signaling; *PLCG1/PLCG2, JAK1*, and *LYN* affect lymphocyte receptor signaling and cytokine responses; and the more recently reported *TRAP1, DPP9, NLRC3*, and *FBXW11* variants provide additional links to mitochondrial, inflammasome, and NF-κB biology. Because evidence for these genes ranges from recurrent functional observations to single-patient reports, they should not be considered equally established disease mechanisms.

### 8.1 IL-1 receptor: unopposed signaling due to selective IL-1Ra resistance

Wang et al. (72) identified a *de novo IL1R1* p.Lys131Glu variant in a patient with chronic recurrent multifocal osteomyelitis (CRMO). The authors combined ligand-binding analyses with cellular and *in vivo* functional studies to determine the molecular consequence of the variant. The p.Lys131Glu substitution altered a positively charged residue within IL-1R1 and selectively impaired interaction with the antagonist IL-1Ra, while preserving receptor recognition of the agonists IL-1α and IL-1β. Consistent with this receptor-level defect, patient PBMCs, particularly monocytes and neutrophils, showed strong inflammatory signatures, supporting persistent IL-1 signaling in the absence of effective IL-1Ra antagonism. In mice carrying the homologous *IL1R1* mutation, the phenotype was reproduced by hyperinflammation, increased susceptibility to collagen antibody-induced arthritis, and pathological osteoclastogenesis. Together, these findings support a mechanism of selective IL-1Ra resistance rather than generalized loss of IL-1R1 ligand binding, resulting in unopposed IL-1α/IL-1β signaling. Leveraging this mechanistic distinction, the authors designed an IL-1 therapeutic that preferentially traps IL-1β and IL-1α while sparing IL-1Ra, illustrating how functional characterization of a disease-associated receptor variant can inform rational drug design.

### 8.2 *IRAK2* and *IKBKB*: NF-κB pathway gain-of-function

Boyd et al. (73) identified a novel homozygous *IRAK2* variant in a child with sJIA complicated by lung disease. THP-1 cells homozygous for the mutation showed increased IL-6, IL-18, and ISG expression after LPS stimulation, recapitulating the clinical pattern. Sacco et al. (74) described a heterozygous gain-of-function *IKBKB* variant (p.Thr559Met) in an infant with autoimmunity and autoinflammation, showing increased basal phosphorylation of IKKα/β and p65.

### 8.3 *PLCG2 and PLCG1*: B-cell and T-cell signaling dysregulation

Baysac et al. (75) functionally classified *PLCG2* variants, identifying both gain-of-function and a novel class of monoallelic loss-of-function causing humoral immune deficiency, autoinflammation, herpes viral susceptibility, and NK cell dysfunction – expanding the PLAID/APLAID spectrum. Tao et al. (76) reported the first germline *PLCG1* gain-of-function variant (p.Ser1021Phe) in a patient with immune dysregulation; transcriptomics revealed enhanced NF-κB and type II IFN in T cells and type I IFN in monocytes. Notably, the concomitant type I interferon signature observed in monocytes indicates substantial overlap with the interferonopathy spectrum discussed in Section 3, illustrating the increasing convergence between adaptive immune dysregulation and innate immune activation.

### 8.4 *JAK1* and *LYN*: kinase activation and inflammatory signaling

Takeichi et al. (77) identified a *JAK1* p.His596Asp variant in a patient with autoinflammatory keratinization disease, liver dysfunction, and autism; knock-in mice recapitulated the phenotype with upregulation of NF-κB. De Jesus et al. (78) identified three unrelated boys with truncating (p.Tyr508Ter, p.Gln507Ter) or missense (p.Tyr508Phe) *LYN* variants causing constitutively active Lyn kinase, leading to neutrophilic cutaneous small vessel vasculitis and liver fibrosis; treatment with dasatinib (a Src kinase inhibitor) resolved liver fibrosis. Louvrier et al. (79) independently reported a *de novo LYN* p.Tyr508His variant in a patient with early-onset systemic inflammation, confirming that variants affecting Tyr508 prevent inhibitory phosphorylation and cause autophosphorylation of Tyr397 and NF-κB activation.

### 8.5 Emerging genes: *TRAP1*, *DPP9, NLRC3*, and *FBXW11*

The following genes should currently be regarded as emerging candidate genes rather than definitively established causes of autoinflammatory disease. Although compelling mechanistic evidence has been reported for individual cases, the available data remain limited, and independent replication together with additional functional validation will be required before these genes can be considered established disease genes.

Standing et al. (80) identified a consanguineous kindred with severe autoinflammation homozygous for *MEFV* p.Ser208Cys and *TRAP1* mutations, plus a fourth unrelated patient with compound heterozygous *TRAP1* mutations alone, suggesting that TRAP1 (a mitochondrial chaperone) loss leads to mitochondrial ROS and elevated IL-18. Wolf et al. (81) reported a *de novo DPP9* p.Arg252Pro mutation causing severe infancy-onset hyperinflammation with HLH; the mutation destabilized DPP9 and failed to restrain NLRP1 and CARD8 inflammasomes in a dominant-negative manner. Koşukcu et al. (82) associated a candidate gene *NLRC3* with autoinflammatory features in one of 11 unclassified AID patients undergoing WES. Hebert et al. (83) performed trio-based WES in 123 sporadic IEI patients and identified a canonical splice site DNV in *FBXW11* in a patient with autoinflammatory disease; functional studies showed defective RNA splicing, increased NF-κB p65 signaling, and elevated IL-1β.

Future confirmation in independent cohorts, segregation analyses, and functional studies will be essential to determine whether these genes represent bona fide monogenic autoinflammatory disorders.

## 9 Oligogenic and modifier findings

Oligogenic inheritance represents one of the most important challenges to a strictly Mendelian interpretation of autoinflammatory disease. Across independent cohorts, patients with systemic autoinflammatory phenotypes frequently carry rare variants in more than one SAID-associated gene. The more consistent observation, therefore, is the co-occurrence of multiple rare variants; the more difficult question is whether these variants jointly cause disease, modify an established monogenic phenotype, or represent coincidental background variation.

Nomani et al. (10) provided evidence for enrichment of combined variants in adult-onset SAID. Among 63 patients, 44 (69.8%) carried NOD2 variants together with variants in *MEFV, NLRP12, NLRP3, or TNFRSF1A*, and these combinations were less frequent in the ARIC control population. Papatheodorou et al. (84) similarly identified *TNFRSF11A* rare variants in 12 of 167 SAID patients, with the variants consistently occurring together with established SAID-associated genes and producing a TRAPS-like phenotype. Beketova et al. (85) further reported multiple digenic and oligogenic combinations in a cohort of 115 patients. The convergence of these observations supports the existence of patients with multilocus genetic burden.

However, the evidence for causality is less secure than the evidence for co-occurrence. In many cases, it remains difficult to determine whether the additional variant contributes materially to disease severity or simply reflects the background frequency of rare variants in patients undergoing broad sequencing. Population structure, variant frequency, segregation, and the absence of functional interaction data can all complicate interpretation (10, 84, 85). The contrasting observation by Rowczenio et al. (86) of a clear dominant *MEFV* p.Pro373Leu variant segregating across four generations, with associated AA amyloidosis, further illustrates that atypical inheritance should not automatically be interpreted as oligogenic disease.

Accordingly, current evidence supports a model in which multilocus variation may modify disease expression in a subset of patients, but does not establish oligogenic inheritance as a general explanation for genetically unresolved autoinflammation. Future studies should distinguish simple variant co-occurrence from demonstrable genetic interaction using large case–control cohorts, family segregation, and functional evidence.

## 10 Genetic testing strategies and diagnostic yield

Advances in next-generation sequencing technologies have markedly improved the genetic diagnosis of autoinflammatory diseases. Current evidence supports a stepwise expansion of genetic testing rather than a single universally optimal platform. Targeted panels remain practical for established monogenic autoinflammatory phenotypes, WES increases diagnostic yield and enables discovery of novel genotype–phenotype associations, and WGS is particularly valuable for non-coding, structural, and deep intronic variants. However, the optimal strategy depends on the suspected mechanism: low-level mosaicism requires sufficiently deep targeted sequencing, whereas unresolved cases with negative coding-region analysis may benefit more from WGS. Thus, sequencing breadth and sequencing depth address different diagnostic problems and should not be treated as interchangeable measures of test performance. Recent studies have highlighted the importance of detecting low-level somatic mosaicism, selecting appropriate sequencing strategies, and integrating functional studies with genetic findings. This section summarizes current diagnostic approaches and their limitations.

Genetic testing strategy for AIDs has evolved with technology. Early targeted panels of 10–25 genes yielded diagnoses in 15–23% of patients (87–89). As panels expanded to 65–166 genes, diagnostic yields increased to 32–38% (85, 90). Whole-exome sequencing (WES) has been evaluated in several studies. Batlle-Masó et al. (91) found likely causative variants in 2/22 patients using clinical exome sequencing. Kosukcu et al. (82) achieved a definite/probable diagnosis in 36% (4/11) of unclassified AID patients via WES. Poker et al. (92) performed WES in 125 pediatric patients and found that analyzing 13 genes gave 16% yield, expanding to 542 IEI genes gave 20.8%, but further WES analysis (including trio) did not increase yield, suggesting that virtual panels may be sufficient for many patients. Price-Kuehne et al. (93) compared targeted gene panel (476 patients, 14.9% yield) to WES (210 patients, 19.5% yield) and found that WES enabled novel genotype-phenotype associations and detected incidental findings in 13.8% of cases, which required careful pre-test counselling. The advantage of trio-based WES was demonstrated by Hebert et al. (83), who identified *de novo* variants (e.g., in *NLRP3*, *RELA*, *FBXW11*) that would have been missed by proband-only sequencing.

For somatic mosaicism, high-depth amplicon sequencing is essential; Kant et al. (94) developed a single-molecule molecular inversion probe assay detecting variants down to 1% VAF. McCreary et al. (95) designed a rapid turnaround panel (25 genes) for severe autoinflammation, returning results within 48 hours – a critical advance for acute presentations. Professional guidelines have incorporated these advances: Shinar et al. (96) published ISSAID/EMQN best practice guidelines, adding *ADA2*, *NOD2*, *PSTPIP1*, and *TNFAIP3* to the core genes and recommending testing for mosaicism and copy-number variants.

## 11 Functional validation and variant interpretation

For VUS reclassification, functional evidence should be interpreted together with established clinical and genetic evidence rather than treated as an independent determinant of pathogenicity. Where reported by the primary study, we therefore indicate whether ACMG/AMP criteria, ClinGen gene-specific specifications, or another explicitly defined framework was used. Functional results are described as supporting evidence for reclassification rather than as sufficient evidence on their own, particularly when the experimental system does not reproduce the endogenous genotype or cellular context.

Accurate interpretation of genetic variants remains one of the greatest challenges in molecular diagnosis of autoinflammatory diseases. Functional studies, segregation analyses, and standardized classification frameworks have become increasingly important for distinguishing pathogenic variants from variants of uncertain significance. This section summarizes recent progress and remaining challenges in variant interpretation.

For NF-κB-regulated disorders, reporter-based assays can directly assess whether candidate variants alter pathway activity. Kadowaki et al. (13) used an NF-κB reporter assay to distinguish functionally abnormal and apparently benign *TNFAIP3* missense variants. In inflammasomopathies, complementary readouts such as caspase-1 activity, ASC speck formation, and IL-1β/IL-18 secretion assess different levels of inflammasome activation. Cosson et al. (40, 41) used a systematic functional framework to separate *NLRP3* variants according to their requirements for priming and activation, while Quach et al. (97) demonstrated the potential of a rapid Caspase-Glo 1 assay for higher-throughput assessment of *NLRP3* gain of function.

Interferonopathies require different functional readouts. Merhar et al. (61) used droplet digital PCR to quantify interferon-inducible gene signatures in a patient with a *PSMB9* VUS, illustrating how pathway-level transcriptional phenotyping can provide evidence relevant to both diagnosis and treatment selection. Similarly, PBMC cytokine profiling distinguished different inflammatory responses among *NLRP12* VUS, indicating that functional effects may be stimulus-dependent rather than constitutive (98). Structural and computational approaches, including the AlphaFold2-based analysis reported for an *NLRP12* variant, may provide additional supporting evidence but should not be considered equivalent to direct functional validation (99, 100).

The broader lesson from these studies is that functional validation should be matched to the proposed mechanism. Reporter assays are most informative for signaling variants, inflammasome assays for inflammasome-associated variants, interferon signatures for proteostasis or nucleic-acid sensing disorders, and localization assays for trafficking defects. No single assay provides a universal measure of pathogenicity.

Importantly, functional abnormality should also be distinguished from formal VUS reclassification. The reported studies demonstrate that functional data can strongly support pathogenic or benign interpretations, but the strength of that evidence depends on assay validity, biological relevance, experimental context, and concordance with genetic and clinical findings. The current literature therefore supports functional testing as an important component of variant interpretation while also highlighting the absence of a standardized assay framework across autoinflammatory disease genes.

Functional assays are presented according to the biological question they address and the experimental system in which they were performed. Functional evidence should be interpreted within the ACMG/AMP framework and, where available, relevant ClinGen specifications. An abnormal assay result does not independently establish pathogenicity. The evidentiary strength of a functional result depends on the biological relevance of the assay, the experimental system, appropriate positive and negative controls, assay validation, reproducibility, and concordance with genetic and clinical evidence. Where patient-derived cells were not specifically identified in the source studies, the experimental system should not be inferred to be a primary patient-cell assay.

## 12 Comparison of top findings and areas of expert disagreement

Because the strength of evidence supporting recently identified disease genes varies considerably, careful evaluation of the available genetic, functional, and clinical data is essential. This section compares the current level of evidence across different discoveries and highlights areas requiring further validation, as summarized in **Table 4**.

**Table 4:** Areas of consensus and ongoing controversy in genetic diagnosis and variant interpretation of autoinflammatory diseases.

Topic	Consensus from literature	Disagreements/controversies	Evidence category	References
Somatic *NLRP3* mosaicism	Somatic *NLRP3* mosaicism has been identified in a substantial proportion of patients with clinically suspected CAPS who lack an identifiable germline *NLRP3* variant. Reported VAFs extend into the low single-digit range, and high-depth sequencing improves detection of low-level mosaic variants. In the studies summarized here, sequencing depths exceeding 1500× were used to detect variants at approximately 2–3% VAF.	The clinical significance of variants below approximately 1% VAF remains uncertain. It is not established whether very-low-level variants represent pathogenic mosaicism, age-related clonal hematopoiesis, or technical artifacts. No universal VAF threshold for clinical reporting has been established.	Multiple independent cohorts with functional evidence; analytical thresholds remain study- and platform-dependent	(6, 7, 29–34)
Non-coding *UBA1* variants	Intronic and splice-altering *UBA1* variants have been reported in patients with a strong VEXAS phenotype who lack canonical exon-3 variants. Molecular and, for selected variants, transcript-level findings support abnormal *UBA1* expression or splicing as a disease mechanism.	The minimum VAF required for reliable detection and clinical interpretation is not standardized. The optimal sequencing strategy for intronic variants and the role of RNA confirmation, particularly for suspected splice-altering variants, remain incompletely defined.	Multiple reports with emerging molecular/RNA support; independent clinical replication remains limited for non-coding variants	(8, 9)
*OTULIN* inheritance and mechanism	Biallelic loss-of-function variants are associated with ORAS. Dominant-negative variants have been associated with an ORAS-like phenotype, while haploinsufficiency has been reported in association with staphylococcal necrosis susceptibility. Functional studies support distinct mechanism-specific effects.	The pathogenic significance of monoallelic missense variants without demonstrated loss-of-function or dominant-negative effects remains uncertain. Whether such variants represent pathogenic alleles, risk modifiers, or tolerated variation requires variant-specific evidence.	Multiple unrelated families/patients with functional validation for major mechanisms; variant-level evidence is heterogeneous	(17–23)
Multilocus rare-variant burden	Rare variants in more than one SAID-associated gene are observed in a subset of patients undergoing broad genetic testing. Such observations support the possibility that genetic background may influence phenotype in some patients.	Co-occurrence does not establish causal oligogenic inheritance. Population stratification, ancestry differences, background rare-variant burden, panel size, ascertainment bias, phase, segregation, and limited functional evidence complicate interpretation. Additional variants may represent modifiers or incidental background variation rather than additional causal alleles.	Multiple cohorts reporting multilocus co-occurrence; causal oligogenic interaction not established	(10, 84–86)
First-line genetic testing strategy	Broader sequencing approaches can increase the diagnostic yield beyond targeted testing, while WES and WGS may identify additional coding and non-coding variants. The optimal testing strategy should depend on the phenotype, suspected mechanism, and analytical performance of the assay.	Stepwise testing versus upfront broad sequencing remains debated. The incremental diagnostic yield, cost-effectiveness, and clinical utility of WES/WGS compared with targeted approaches have not been established uniformly across autoinflammatory diseases.	Multiple diagnostic studies for sequencing yield; comparative clinical utility and cost-effectiveness remain limited	(82, 83, 85, 87, 89–96)
Functional testing for VUS	Functional assays can provide important mechanistic evidence for selected variants in genes such as *TNFAIP3*, *NLRP3*, *OTULIN*, and *PSMB9*. The value of functional evidence depends on the biological relevance and validation of the assay.	No universal gold-standard assay exists across SAID genes. Assay systems, controls, readouts, and reproducibility vary substantially. Functional evidence alone does not establish pathogenicity and should be integrated with population, clinical, segregation, computational, RNA, replication, and gene–disease validity evidence according to ACMG/AMP and relevant ClinGen specifications.	Multiple functional assay classes with gene-specific validation; standardized interpretation remains incomplete	(13, 41, 60, 96, 97)
*PSTPIP1* variant pathogenicity	Multiple rare *PSTPIP1* variants have been reported in patients with PAPA, PAMI, and related phenotypes. Functional and clinical analyses indicate substantial heterogeneity among reported variants, with only a subset supported as pathogenic under the criteria applied in the cited study.	The pathogenicity of many individual variants remains uncertain. The classification of *PSTPIP1* variants should not rely solely on functional abnormalities or on the proportion of variants classified as pathogenic in a single cohort. Variant-level interpretation requires integration of segregation, phenotype, population data, functional evidence, and other relevant ACMG/AMP/ClinGen criteria.	Large patient/cohort analysis with selected functional validation; variant-level evidence remains heterogeneous	(51–53)

To avoid subjective numerical grading, evidence strength is described using qualitative evidence categories based on the type, replication, and independence of the available genetic, clinical, and functional evidence. These categories include multiple independent cohorts, replicated case series or unrelated families, single-family reports with functional validation, single-case reports with functional validation, and preclinical or computational evidence only.

## 13 Major open questions and future directions

The synthesis above indicates that the major gaps in autoinflammatory disease genetics are no longer limited to gene discovery. Instead, the key challenges are determining the clinical significance of low-level mosaicism, establishing reproducible standards for functional variant interpretation, distinguishing oligogenic causality from coincidental variant burden, defining the contribution of non-coding variation, and translating genotype–mechanism relationships into treatment selection. These priorities follow directly from the areas in which current evidence is either inconsistent across studies or supported mainly by small cohorts and single reports.

### 13.1 Open questions (from literature gaps)

1. What is the true prevalence of non-coding and intronic variants in AIDs? Most diagnostic pipelines still focus on exons; systematic WGS in large undiagnosed cohorts is needed (8).
2. How can functional validation be standardized across laboratories? A ClinGen-style variant curation expert panel for AID genes is urgently needed, with agreed-upon assays and thresholds (13, 41).
3. What is the clinical significance of extremely low-level mosaicism (<1% VAF)? Longitudinal studies and single-cell DNA/RNA sequencing are required to distinguish pathogenic mosaicism from CHIP (31, 33).
4. How often is oligogenic inheritance truly causal vs. polygenic risk? Large-scale case-control studies (e.g., using gnomAD) and segregation analyses in extended families are needed (10).
5. Can treatment response be predicted from genotype? Few prospective genotype-driven treatment trials exist. For example, JAK inhibitors work in some interferonopathies but not all; IL-1 blockers are effective in CAPS but not in all *NLRP3* variants.
6. What is the natural history of somatic mosaicism over decades? Kato et al. (33) showed that some mosaic VAFs increase over time due to clonal hematopoiesis. Long-term follow-up is required.

### 13.2 Future directions (perspectives)

- Long-read sequencing (PacBio, Oxford Nanopore) will likely become routine to detect structural variants, repeat expansions, and deep intronic variants missed by short-read WGS.
- Single-cell multi-omics (DNA + RNA + protein) will help dissect how mosaic variants drive inflammation in specific lineages.
- Artificial intelligence for variant prioritization and genotype-phenotype matching, using protein structure (AlphaFold) and pathway signatures, is an emerging approach (99, 100).
- Targeted therapies such as STING inhibitors (H-151, amlexanox) are being explored in preclinical models (58); gene editing for somatic variants remains hypothetical.
- Newborn screening for actionable AIDs using dried blood spot NGS is a potential long-term goal.

## 14 Limitations of current evidence

Several limitations constrain the strength of current conclusions in autoinflammatory disease genetics. First, much of the emerging literature remains based on individual families, case reports, or small cohorts, limiting independent replication and the ability to establish robust genotype–phenotype relationships. This is particularly relevant for recently proposed disease genes and for rare variant classes for which only one or a few affected individuals have been described.

Second, functional evidence is heterogeneous. Although functional assays can provide important support for variant interpretation, different studies use different cellular systems, stimulation conditions, readouts, and thresholds, making direct comparison difficult (13, 41). A functional abnormality demonstrated in an overexpression system may also have a different evidentiary weight from a phenotype reproduced in primary patient cells. Consequently, functional findings should be interpreted in the context of the experimental system and integrated with genetic and clinical evidence.

Third, interpretation of somatic mosaicism remains limited by differences in sequencing sensitivity, tissue sampling, and longitudinal follow-up. Low-level variants may be missed by conventional sequencing, whereas very low VAF variants can be difficult to distinguish from age-related clonal hematopoiesis (31, 33). The absence of standardized reporting of sequencing depth, cellular distribution, and analytical sensitivity further complicates comparisons between cohorts.

Fourth, the causal interpretation of oligogenic findings remains uncertain. Although enrichment of multiple rare variants has been reported in several cohorts, population stratification and background rare-variant burden can confound the interpretation of multilocus genotypes (10). Finally, long-term clinical outcomes and genotype–treatment response relationships remain insufficiently characterized. These limitations indicate that future progress will depend not only on additional gene discovery but also on larger prospective cohorts, standardized functional validation, longitudinal mosaicism studies, and systematic integration of genetic, mechanistic, and clinical evidence.

## 15 Conclusion

Recent advances have transformed the genetic landscape of autoinflammatory disease from a predominantly Mendelian framework into a broader spectrum encompassing somatic mosaicism, non-coding variation, oligogenic effects, and defects in ubiquitination, inflammasome activation, interferon signaling, intracellular trafficking, metabolism, and programmed cell death. Across these pathways, several recurring principles emerge: different genetic lesions can converge on the same inflammatory output, identical genes can produce different phenotypes through distinct molecular mechanisms, and the clinical significance of a variant depends increasingly on its cellular context, allelic mechanism, and functional consequence.

The strongest evidence currently supports low-level *NLRP3* mosaicism as an important cause of late-onset CAPS, non-coding *UBA1* variation as an extension of the VEXAS spectrum, distinct *OTULIN* inheritance mechanisms, and several newly established interferon and trafficking disorders (8, 17–23, 31, 57–59). In contrast, the causal significance of very low-level mosaicism, multilocus rare-variant combinations, and several emerging candidate genes remains less certain. Functional studies have become central to resolving these uncertainties, but their interpretation is limited by heterogeneous experimental systems and the absence of standardized assay frameworks.

Future progress will therefore depend less on simply identifying additional variants than on integrating sequencing sensitivity, cellular distribution, functional evidence, longitudinal observation, and clinical outcomes into a unified framework. Such an approach should improve both diagnostic accuracy and mechanistic understanding while allowing the growing number of genomic findings in autoinflammatory disease to be distinguished according to the strength of the evidence supporting them.

## Glossary

AID: Autoinflammatory disease
CAPS: Cryopyrin-associated periodic syndrome
CANDLE: Chronic atypical neutrophilic dermatosis with lipodystrophy and elevated temperatures
CHIP: Clonal hematopoiesis of indeterminate potential
COPI: Coat protein complex I
CRIA: Cleavage-resistant RIPK1-induced autoinflammatory syndrome
CRMO: Chronic recurrent multifocal osteomyelitis
ddPCR: Digital droplet polymerase chain reaction
DNV: *De novo* variant
DUB: Deubiquitinase
ER: Endoplasmic reticulum
FCAS: Familial cold autoinflammatory syndrome
FCAS2: Familial cold autoinflammatory syndrome type 2
GOF: Gain-of-function
HA20: Haploinsufficiency of A20
HIDS: Hyper-immunoglobulin D syndrome
HLH: Hemophagocytic lymphohistiocytosis
HSCT: Hematopoietic stem cell transplantation
IEI: Inborn errors of immunity
IFN: Interferon
IgD: Immunoglobulin D
IL: Interleukin
iPSC: Induced pluripotent stem cell
ISG: Interferon-stimulated gene
LOF: Loss-of-function
LRR: Leucine-rich repeat
LUBAC: Linear ubiquitin chain assembly complex
MAS: Macrophage activation syndrome
MKD: Mevalonate kinase deficiency
NAIAD: NLRP1-associated autoinflammation with arthritis and dyskeratosis
NBD: Nucleotide-binding domain
NDAS: NEMO deleted exon 5 autoinflammatory syndrome
NEMO: NF-κB essential modulator
NGS: Next-generation sequencing
NOCARH: Neonatal-onset cytopenia, autoinflammation, rash, and hemophagocytic lymphohistiocytosis
NOMID: Neonatal-onset multisystem inflammatory disease
ORAS: OTULIN-related autoinflammatory syndrome
PAMI: PSTPIP1-associated myeloid-related inflammatory syndrome
PAPA: Pyogenic arthritis, pyoderma gangrenosum, and acne
PBMC: Peripheral blood mononuclear cell
PLAID: PLCγ2-associated antibody deficiency and immune dysregulation
PRAAS: Proteasome-associated autoinflammatory syndromes
ROS: Reactive oxygen species
SAID: Systemic autoinflammatory disease
SAVI: STING-associated vasculopathy with onset in infancy
sJIA: Systemic juvenile idiopathic arthritis
SLE: Systemic lupus erythematosus
TGP: Targeted gene panel
TRAPS: TNF receptor-associated periodic syndrome
VAF: Variant allele frequency
VEXAS: Vacuoles, E1 enzyme, X-linked, autoinflammatory, somatic
VUS: Variant of uncertain significance
WES: Whole-exome sequencing
WGS: Whole-genome sequencing
