# Supplementary material for: Recent variant discoveries and emerging genetic mechanisms in autoinflammatory diseases
Source: Front Immunol. 2026 Sep 15;17:1910164. doi: 10.3389/fimmu.2026.1910164 (PMC13620470; doi:10.3389/fimmu.2026.1910164)
Supplement: Supplementary file 1 [file Table1.docx]

**Supplementary Table S1. Database-specific literature search strategies and eligibility criteria**

| **Database** | **Search period** | **Search strategy** | **Limits** |
| --- | --- | --- | --- |
| **PubMed/MEDLINE – Core search** | January 1, 2014–March 31, 2026 | ("Autoinflammatory Diseases"[Mesh] OR autoinflamm*[Title/Abstract] OR "systemic autoinflammatory disease*"[Title/Abstract] OR SAID[Title/Abstract]) AND (genetic*[Title/Abstract] OR variant*[Title/Abstract] OR mutation*[Title/Abstract] OR mosaic*[Title/Abstract] OR somatic[Title/Abstract] OR "gain-of-function"[Title/Abstract] OR "loss-of-function"[Title/Abstract] OR oligogenic[Title/Abstract] OR "rare variant*"[Title/Abstract] OR "functional validation"[Title/Abstract] OR "genetic testing"[Title/Abstract]) | English; publication date 2014/01/01–2026/03/31 |
| **PubMed/MEDLINE – Targeted gene/pathway search** | January 1, 2014–March 31, 2026 | ((UBA1 OR OTULIN OR SHARPIN OR TNFAIP3 OR NFKB1 OR IKBKG OR NLRP3 OR NLRC4 OR NLRP1 OR PSTPIP1 OR CASP1 OR TMEM173 OR TBK1 OR ARF1 OR REXO2 OR COPA OR PSMB8 OR PSMB9 OR PSMB10 OR PSMA5 OR PSMC5 OR CDC42 OR PMVK OR RIPK1 OR RIPK3 OR IL1R1 OR IRAK2 OR IKBKB OR PLCG1 OR PLCG2 OR JAK1 OR LYN OR TRAP1 OR DPP9 OR NLRC3 OR FBXW11) AND (autoinflamm*[Title/Abstract] OR "autoinflammatory disease*"[Title/Abstract] OR interferonopathy[Title/Abstract] OR inflammasom*[Title/Abstract] OR VEXAS[Title/Abstract] OR CAPS[Title/Abstract] OR TRAPS[Title/Abstract] OR PRAAS[Title/Abstract] OR NOCARH[Title/Abstract])) | English; publication date 2014/01/01–2026/03/31 |
| **PubMed/MEDLINE – Somatic mosaicism** | January 1, 2014–March 31, 2026 | ((autoinflamm*[Title/Abstract] OR CAPS[Title/Abstract] OR VEXAS[Title/Abstract] OR TRAPS[Title/Abstract]) AND ("somatic mosaicism"[Title/Abstract] OR mosaic*[Title/Abstract] OR VAF[Title/Abstract] OR "variant allele frequency"[Title/Abstract])) | English; publication date 2014/01/01–2026/03/31 |
| **PubMed/MEDLINE – Functional validation / VUS** | January 1, 2014–March 31, 2026 | ((autoinflamm*[Title/Abstract] OR SAID[Title/Abstract]) AND (VUS[Title/Abstract] OR "variant of uncertain significance"[Title/Abstract] OR "functional validation"[Title/Abstract] OR pathogenicity[Title/Abstract] OR ACMG[Title/Abstract])) | English; publication date 2014/01/01–2026/03/31 |
| **PubMed/MEDLINE – Genetic testing** | January 1, 2014–March 31, 2026 | ((autoinflamm*[Title/Abstract] OR "autoinflammatory disease*"[Title/Abstract]) AND ("targeted gene panel"[Title/Abstract] OR "whole exome sequencing"[Title/Abstract] OR WES[Title/Abstract] OR "whole genome sequencing"[Title/Abstract] OR WGS[Title/Abstract] OR "genetic testing"[Title/Abstract] OR "next generation sequencing"[Title/Abstract])) | English; publication date 2014/01/01–2026/03/31 |
| **Web of Science Core Collection – Core search** | January 1, 2014–March 31, 2026 | TS=(autoinflamm* AND (genetic* OR variant* OR mutation* OR mosaic* OR somatic OR "gain-of-function" OR "loss-of-function" OR oligogenic OR "rare variant*" OR "functional validation" OR "genetic testing")) | English; publication year 2014–2026 |
| **Web of Science Core Collection – Targeted search** | January 1, 2014–March 31, 2026 | TS=((UBA1 OR OTULIN OR SHARPIN OR TNFAIP3 OR NFKB1 OR IKBKG OR NLRP3 OR NLRC4 OR NLRP1 OR PSTPIP1 OR CASP1 OR TMEM173 OR TBK1 OR ARF1 OR REXO2 OR COPA OR CDC42 OR PMVK OR RIPK1 OR RIPK3 OR IL1R1 OR IRAK2 OR IKBKB OR PLCG1 OR PLCG2 OR JAK1 OR LYN OR TRAP1 OR DPP9 OR NLRC3 OR FBXW11) AND autoinflamm*) | English; publication year 2014–2026 |
| **Scopus – Core search** | January 1, 2014–March 31, 2026 | TITLE-ABS-KEY(autoinflamm* AND (genetic* OR variant* OR mutation* OR mosaic* OR somatic OR "gain-of-function" OR "loss-of-function" OR oligogenic OR "rare variant*" OR "functional validation" OR "genetic testing")) | English; publication year 2014–2026 |
| **Scopus – Targeted search** | January 1, 2014–March 31, 2026 | TITLE-ABS-KEY((UBA1 OR OTULIN OR SHARPIN OR TNFAIP3 OR NFKB1 OR IKBKG OR NLRP3 OR NLRC4 OR NLRP1 OR PSTPIP1 OR CASP1 OR TMEM173 OR TBK1 OR ARF1 OR REXO2 OR COPA OR CDC42 OR PMVK OR RIPK1 OR RIPK3 OR IL1R1 OR IRAK2 OR IKBKB OR PLCG1 OR PLCG2 OR JAK1 OR LYN OR TRAP1 OR DPP9 OR NLRC3 OR FBXW11) AND autoinflamm*) | English; publication year 2014–2026 |

**Study selection criteria.** Eligible publications included original research articles, cohort studies, case series, case reports, genetic studies, and mechanistic/functional studies addressing genetic mechanisms, variant interpretation, somatic mosaicism, or genetic diagnosis of systemic autoinflammatory disease. Conference abstracts and preprints were retained only when they contained directly relevant primary data and no corresponding full-text peer-reviewed publication was identified during the search period. Reviews, consensus statements, and guidelines were used for contextual information and reference-list screening but were not considered primary evidence. Publications unrelated to systemic autoinflammatory disease, lacking relevant genetic or mechanistic information, or unavailable in English were excluded.

**Search supplementation.** Reference lists of relevant primary articles and selected reviews were screened manually to identify additional publications not retrieved by the electronic searches.
